# Unveiling the proteome-wide autoreactome enables enhanced evaluation of emerging CAR T cell therapies in autoimmunity

**DOI:** 10.1172/JCI180012

**Published:** 2024-05-16

**Authors:** Aaron Bodansky, David J.L. Yu, Alysa Rallistan, Muge Kalaycioglu, Jim Boonyaratanakornkit, Damian J. Green, Jordan Gauthier, Cameron J. Turtle, Kelsey Zorn, Brian O’Donovan, Caleigh Mandel-Brehm, James Asaki, Hannah Kortbawi, Andrew F. Kung, Elze Rackaityte, Chung-Yu Wang, Aditi Saxena, Kimberly de Dios, Gianvito Masi, Richard J. Nowak, Kevin C. O’Connor, Hao Li, Valentina E. Diaz, Rowan Saloner, Kaitlin B. Casaletto, Eva Q. Gontrum, Brandon Chan, Joel H. Kramer, Michael R. Wilson, Paul J. Utz, Joshua A. Hill, Shaun W. Jackson, Mark S. Anderson, Joseph L. DeRisi

**Affiliations:** 1 Department of Pediatrics, Division of Critical Care, and; 2 Diabetes Center, School of Medicine, UCSF, San Francisco, California, USA.; 3 Department of Medicine, Division of Immunology and Rheumatology, and; 4 Institute of Immunity, Transplantation, and Infection, Stanford University, Stanford, California, USA.; 5 Fred Hutchinson Cancer Center, Seattle, Washington, USA.; 6 University of Washington School of Medicine, Seattle, Washington, USA.; 7 Department of Biochemistry and Biophysics,; 8 Biomedical Sciences Program,; 9 Medical Scientist Training Program, and; 10 Biological and Medical Informatics Program, UCSF, San Francisco, California, USA.; 11 Chan Zuckerberg Biohub San Francisco, San Francisco, California, USA.; 12 Department of Neurology, Yale School of Medicine, New Haven, Connecticut, USA.; 13 Department of Immunobiology, School of Medicine, Yale University, New Haven, Connecticut, USA.; 14 Memory and Aging Center, Department of Neurology, Weill Institute for Neurosciences,; 15 Weill Institute for Neurosciences, and; 16 Department of Neurology, UCSF, San Francisco, California, USA.; 17 Laboratory Medicine and Pathology, University of Washington School of Medicine, Seattle, Washington, USA.; 18 Seattle Children’s Research Institute, Seattle, Washington, USA.; 19 Department of Pediatrics, University of Washington School of Medicine, Seattle, Washington, USA.

**Keywords:** Autoimmunity, Therapeutics, Adaptive immunity, Autoimmune diseases, Immunotherapy

## Abstract

Given the global surge in autoimmune diseases, it is critical to evaluate emerging therapeutic interventions. Despite numerous new targeted immunomodulatory therapies, comprehensive approaches to apply and evaluate the effects of these treatments longitudinally are lacking. Here, we leveraged advances in programmable-phage immunoprecipitation methodology to explore the modulation, or lack thereof, of autoantibody profiles, proteome-wide, in both health and disease. Using a custom set of over 730,000 human-derived peptides, we demonstrated that each individual, regardless of disease state, possesses a distinct and complex constellation of autoreactive antibodies. For each individual, the set of resulting autoreactivites constituted a unique immunological fingerprint, or “autoreactome,” that was remarkably stable over years. Using the autoreactome as a primary output, we evaluated the relative effectiveness of various immunomodulatory therapies in altering autoantibody repertoires. We found that therapies targeting B cell maturation antigen (BCMA) profoundly altered an individual’s autoreactome, while anti-CD19 and anti-CD20 therapies had minimal effects. These data both confirm that the autoreactome comprises autoantibodies secreted by plasma cells and strongly suggest that BCMA or other plasma cell–targeting therapies may be highly effective in treating currently refractory autoantibody-mediated diseases.

## Abstract 2

Proteome-wide antibody profiling technology identifies the presence of a unique set of autoantibodies per individual — the autoreactome — that could be targeted in autoimmunity.

## Introduction

Autoantibodies have been identified in a wide range of autoimmune diseases (1–4). In many cases these autoantibodies are directly pathogenic (5–10), while in others they amplify or support T cell–driven pathologies (6, 11). Numerous technologies now allow for the detection of autoantibodies targeting many proteins simultaneously (12–15), and in the case of phage immunoprecipitation and sequencing (PhIP-Seq), the entire human proteome (16), using relatively small amounts of plasma or serum. Using these new tools, a wide spectrum of putatively novel autoantibodies have been discovered to be associated with various disease states (11, 17–21), including, but not limited to, paraneoplastic encephalitis, lipodystrophy, inborn genetic disorders, and multisystem inflammatory syndrome in children (11, 16–23). To accurately identify shared autoreactive profiles among individuals that also share a disease state, we and others have found that serum samples from large numbers of healthy individuals are required, due to the presence of highly diverse autoreactivities present in every individual (17). However, it remains unclear whether individual proteome-wide autoreactive signatures are stable or variable with time or with immunosuppressive treatment. Here, we leveraged a custom proteome-wide PhIP-Seq autoantibody discovery platform to comprehensively profile the autoreactive repertoire in healthy individuals and discovered that each individual harbors a unique, distinctive, and highly reproducible set of autoreactivities that we term the “autoreactome.” Using longitudinal samples from an additional cohort of healthy individuals, we determined that an individual’s autoreactome, once formed, remains minimally changed over the course of years.

Extending these findings, we explored the effect of B cell depletion therapies upon the autoreactome. Various surface markers are expressed and then downregulated over the course of B cell development and maturation (24, 25). A subset of these surface markers are targeted by B cell–depleting therapies that are used to treat suspected autoantibody-mediated diseases, yet a comprehensive evaluation of the effects of these treatments on autoantibodies proteome-wide remains lacking. The most commonly used agent, rituximab, targets CD20, which is not expressed by antibody-secreting cells (26). Recently, chimeric antigen receptor T (CAR T) cell therapy targeting CD19^+^ cells was shown to be safe and effective in the treatment of refractory systemic lupus erythematosus (SLE) (27). CD19 is expressed on naive and memory B cells, with reduced expression on plasmablasts and plasma cells (28, 29). CAR T cells targeting B cell maturation antigen (BCMA), which is expressed primarily by antibody-secreting plasma cells (30, 31), are approved for the treatment of multiple myeloma (32, 33). Here, we examined the impact of three major B cell–depleting therapies, rituximab (anti-CD20), anti-CD19 CAR T cells, and anti-BCMA CAR T cells by PhIP-Seq and validate our findings using orthogonal assays. We demonstrate profound impact of anti-BCMA targeted therapies and minimal impact of therapies targeting CD19 or CD20 on individuals’ autoantibody signatures.

## Results

### Healthy individuals harbor a unique set of autoreactivities: the autoreactome.

The rich history of “natural autoantibody” biology has shown that many healthy individuals share a common set of autoreactive antibodies (34–36). This work has recently been scaled to detect shared autoantibodies across thousands of proteins and has identified a set of autoantibodies present in many individuals, which some have termed the “autoantibodyome” (37). It is also known that specific disease-associated autoantibodies are often present in certain healthy individuals, and recent efforts have shown that different individuals harbor distinctive patterns of reactivity to these known autoantigens. Using our customized, previously described 730,000-element PhIP-Seq (11, 17–21, 38) we are able to build considerably on this previous work by comprehensively and quantitatively characterizing in high resolution the proteome-wide set of autoreactivities distinctive of each individual (hereafter referred to as the “autoreactome”), revealing a new appreciation for the unique diversity each individual harbors.

**Figure 1:**
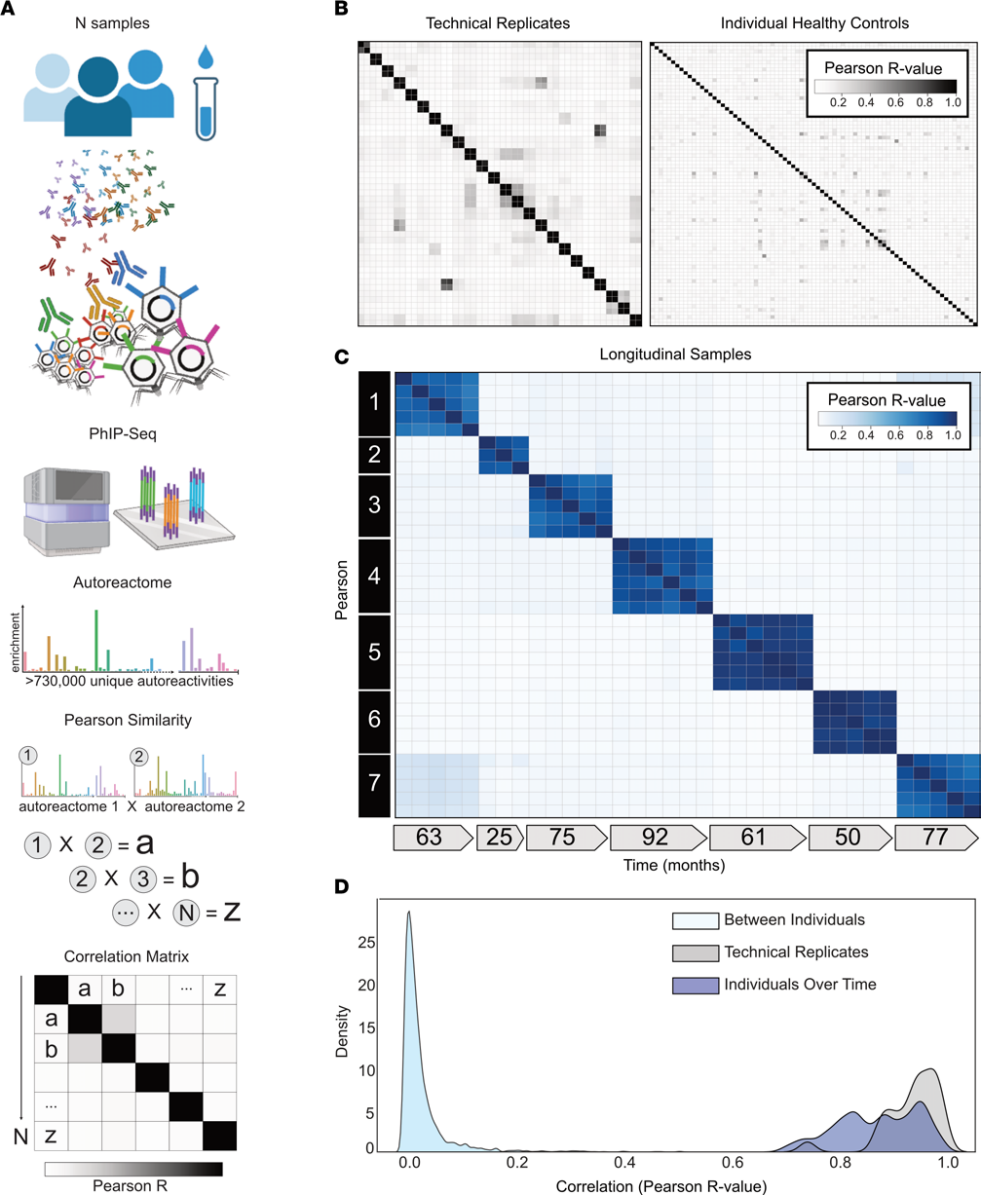
Individuals harbor a unique set of longitudinally stable autoreactivities. (**A**) Graphical representation of the analytical approach to quantitatively compare individual samples’ unique proteome-wide autoantibody signal (“Autoreactome,” containing up to 730,000 unique autoreactivities) to any other sample. (**B**) Correlation matrices showing Pearson’s correlation coefficients of complete PhIP-Seq signal in healthy individuals. Left: 48 samples representing 24 individuals in technical replicate (same data set as used in **A**). Right: 79 distinct individuals. (**C**) Correlation matrix showing Pearson’s *r* values for complete PhIP-Seq enrichment in 7 distinct individuals, each of whom have serial samples over at least 3 years. (**D**) Kernel density estimate plot showing distribution of Pearson’s *r* correlation coefficients among technical replicates, individuals over time, and between different individuals.

To define the variation among autoreactomes of healthy individuals we performed PhIP-Seq and analyzed resulting data from 79 healthy blood donor samples collected prior to the COVID-19 pandemic (healthy demographics in Supplemental Table 1; supplemental material available online with this article; https://doi.org/10.1172/JCI180012DS1) and developed an analytical approach for quantitatively assessing the similarity of autoreactomes among different samples (Figure 1A). For a robust quantitative analysis of inter- and intraindividual similarity by PhIP-Seq, results must be highly reproducible, as measured by technical replicate. Over the past several years, we have refined our PhIP-Seq protocols to maximize reproducibility. The master version of this protocol is available at Protocols.io (https://www.protocols.io/view/derisi-lab-phage-immunoprecipitation-sequencing-ph-czw7x7hn?step=14.1) and is listed as “DeRisi Lab Phage Immunoprecipitation and Sequencing.” PhIP-Seq protocol performance was evaluated by identifying the PhIP-Seq enrichment similarity of 48 samples representing 24 sets of technical replicates. The relative amounts of each phage-presented peptide that were immunoprecipitated within a given sample were calculated and then used to compare each individual sample. Technical replicates showed high reproducibility (Pearson’s *r* coefficient median = 0.946; first quartile [Q1] = 0.907 and third quartile [Q3] = 0.974) (Figure 1B, left). Next, the similarity of individual autoreactomes was compared among each of the 79 healthy individuals in this study. For each sample, the PhIP-Seq enrichment was compared with each of the 78 other samples. We found that individual autoreactomes were distinctive, with very little similarity to others (Pearson’s *r* coefficient median = 0.021; Q1 = 0.018, Q3 = 0.023) (Figure 1B, right). These results demonstrate that each individual harbors a highly reproducible and unique autoreactome, comprising the relative signal in up to 730,000 different autoreactivities. Having established the uniqueness of individual autoreactomes, we next sought to determine whether those specific autoantibodies that were most enriched within an individual might have a higher degree of sharing. For each of the 79 individuals in our cohort, the top 10 autoantibodies were calculated (fold change [FC] over mock IP), which yielded a total of 623 unique autoantigen targets. Of these, 286 of the autoantibodies were unique to the individual in whom they were initially identified, and the mean number of individuals who shared an autoantibody was 2.53 of 79 (3.2%) (Supplemental Figure 1A). The cellular locations of the 623 autoantigens were analyzed by gene ontology analysis, and the vast majority were noted to intracellular antigens, consistent with the notion that the identified autoantibodies in these healthy individuals were unlikely to be pathologic (Supplemental Figure 1B). However, a small subset of the autoantibody targets are predicted to be extracellular or cell surface proteins, suggesting that under certain conditions some of these autoantibodies could hypothetically have biological effects.

### The autoreactome is longitudinally stable.

While individual autoreactomes appear distinct, it remained an open question whether these profiles were stable over time within a given individual, which has been previously shown for certain individual autoantibodies (39). To address this question, we performed PhIP-Seq on longitudinal serum samples from 7 distinct healthy individuals collected over a median of 63 months (35 samples total; Q1 = 55.5 months, Q3 = 76 months) (longitudinal demographics in Supplemental Table 1). None of these individuals were being treated with immunomodulatory agents at any point during sample collection; however, 1 had basal cell carcinoma and another had a history of Sjögren’s disease (clinical details in Supplemental Table 2). We compared the complete PhIP-Seq enrichment profile for each sample to all other samples (Figure 1C). These results clearly revealed that the intraindividual autoreactome profiles were highly correlated (Pearson’s *r* coefficient median = 0.883; Q1 = 0.817, Q3 = 0.940). Conversely, the autoreactome of longitudinal samples within an individual was significantly more similar to each other than to the autoreactomes between different individuals (Mann-Whitney *U*, *P* = 4.91 × 10^–95^). Additionally, the distribution of intraindividual correlations overlapped considerably with the distribution from technical replicates (104 of 146, 71.2%, longitudinal sample *r* values fall within 2 standard deviations of the mean of technical replicate *r* values), indicating that in most cases longitudinal autoreactomes are as similar to one another as technical replicates (Figure 1D). These results suggest that the dominant humoral determinants of autoreactivity within an individual are not subject to large variation by this assay, at least within the median 5-year time scale of this analysis.

### The autoreactome is minimally altered by IVIG.

We and others have used PhIP-Seq to investigate autoimmune disease determinants; however, many patients with immune disorders or deficiencies are treated with intravenous immunoglobulin (IVIG). IVIG is pooled from thousands of donors (40) and therefore may contain relatively common autoantibodies, which have the potential to confound autoantibody assays (41). To investigate the effect of IVIG on intraindividual PhIP-Seq performance, we examined a cohort of 189 samples from patients with myasthenia gravis, an autoantibody-mediated autoimmune disease. Among these 189 samples we identified 4 paired sets of samples in which the first collection was in a patient naive to any immunomodulatory treatments, the second sample was within 6 weeks of IVIG treatment (2 within days, 1 within 3 weeks, 1 within 6 weeks), and no additional immunomodulatory treatments had been given except for steroids and in 1case azathioprine (IVIG demographics in Supplemental Table 1; sample details in Supplemental Table 3).

PhIP-Seq was performed on these samples, and the mean correlation before and after IVIG was 0.815 (relative to 0.87 in longitudinal samples from individuals over time without any intervention) (Supplemental Figure 2, A and B). To further determine whether there were directional differences in the levels of PhIP-Seq detected autoantibodies following IVIG treatment, the sum of the top 10 differentially enriched autoantibodies (see Methods) derived from each individual before and after IVIG administration was compared. No significant difference was observed (2-sided paired-sample Wilcoxon’s test, *P* = 0.625) (Supplemental Figure 2C) before and after IVIG.

### Evaluation of B cell depleting therapies on autoantibody repertoires.

It is widely known that rituximab (anti-CD20) and anti-CD19 therapies can reduce the levels of certain individual autoantibodies (27, 42, 43). Previous work has suggested that BCMA-directed therapy may decrease in vivo levels of pathogen-specific antibodies or specific autoantibodies, but the published data are limited (43, 44). The relative effects of different immunomodulatory therapies on proteome-wide autoantibody repertoires remain unknown. This information, however, is required to overcome the limitations which have prevented others from describing longitudinal autoantibody changes in plasma cell–depleting therapies. These barriers include an inability to identify sufficient numbers of pretreatment autoantibodies in the samples to track longitudinal changes attributable to therapy (43) and the near-universal use of posttreatment IVIG, which cannot be accounted for with conventional assays and, therefore, limits studies to cross-sectional cohorts representing a single time point “snapshot” of antibody levels (44). By defining the autoreactome and tracking longitudinal changes following immunomodulatory treatments, we overcome these previous limitations to provide a quantitative assessment of autoantibody changes attributable to a specific intervention.

### Rituximab treatment has minimal effect on the autoreactome.

Depletion of CD20^+^ B cells with rituximab is a common treatment in autoimmunity and presumed autoantibody-mediated diseases (45–47). To determine the extent to which rituximab treatment alters the autoreactome, we examined our cohort of 189 samples from patients with myasthenia gravis to identify pairs of pre- and postrituximab treatment samples. Like other autoantibody-mediated diseases, treatment of myasthenia gravis can include multiple concurrent therapies that could potentially alter the autoantibody profile of an individual. To be conservative, we excluded all patients who had received any immunomodulation (including IVIG and plasma exchange) other than rituximab, steroids, or azathioprine and for whom a pretreatment sample was not available. Using these stringent criteria, 35 longitudinal samples were identified from 7 individuals and analyzed by PhIP-Seq (rituximab demographics in Supplemental Table 1; sample clinical details in Supplemental Table 4).

**Figure 2:**
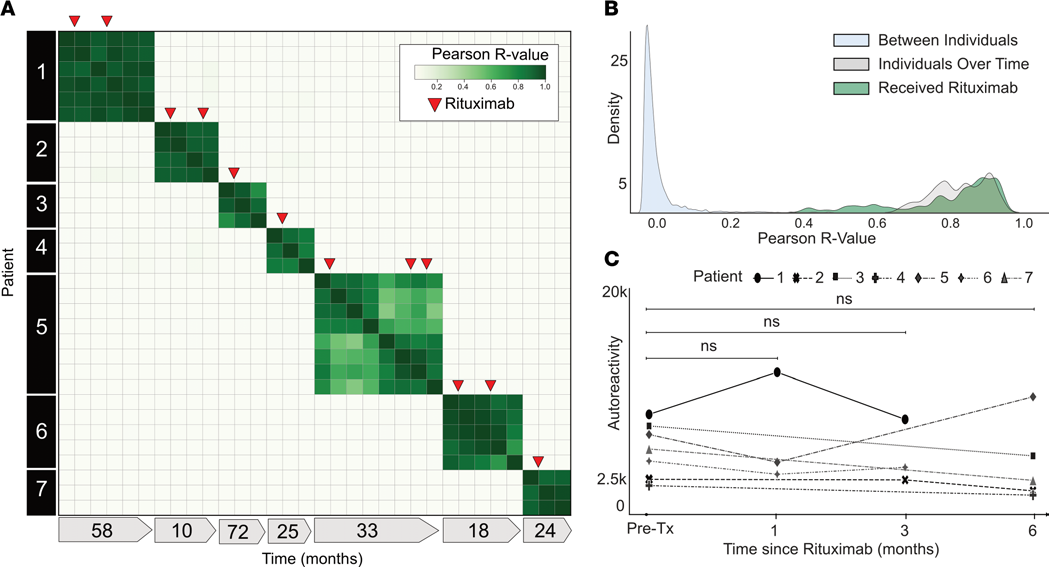
Rituximab treatment does not significantly alter the autoreactome. (**A**) Correlation matrix showing Pearson’s *r* values of complete PhIP-Seq signal in 7 distinct individuals with myasthenia gravis, each of whom were either rituximab naive or had not received rituximab for more than 6 months prior to first sample collection. Red arrows represent administration of rituximab. (**B**) Kernel density estimate plot showing distribution of Pearson’s *r* value correlation coefficients among longitudinal samples from individuals receiving rituximab relative to longitudinal samples from individuals received no intervention. (**C**) Line plots showing the autoreactivity (sum of top 10 PhIP-Seq *Z* scores relative to the 79 individuals acting as healthy controls) for each patient over the first 6 months following the initial rituximab dose. One-way paired-sample Wilcoxon’s test, *P* = 0.625 at 1 month, 0.125 at 3 months, and 0.3125 at 6 months.

The PhIP-Seq enrichment profile for each sample from a given individual who received rituximab was compared. Despite rituximab therapy, the autoreactome remained stable overall within each individual over time (Pearson’s *r* coefficient mean of 0.887; Q1 = 0.782, Q3 = 0.940) (Figure 2A). The overall distribution of correlation coefficients from individuals over time who received rituximab was similar to that of individuals who did not receive rituximab (108 of 136, 79.4%, within 2 standard deviations of the mean of *r* values from longitudinal samples without interventions) and was not significantly different (Mann-Whitney *U* test, *P* = 0.66) (Figure 2B). Specimens from patient 5 (time points 5–8) were the only instances in which the autoreactome changed greater than would be expected from time alone. At the time point with the greatest change in the autoreactome (time point 5), this patient was noted to be minimally symptomatic. However, given that this represents a single time point in a single patient, it is difficult to drawn meaningful conclusions. While rituximab is known to transiently reduce certain antibodies, these results suggest the overall profile of autoreactivity after rituximab treatment remains essentially unchanged.

To determine whether there were decreases in subsets of PhIP-Seq–enriched autoreactivities following rituximab treatment, as opposed to the complete profile, the sum of the top 10 differentially enriched protein targets (see Methods) in each individual was calculated at the time of initial sample collection and then tracked longitudinally following the first dose of rituximab. To avoid overlapping timelines, data points following an additional round of rituximab therapy were removed from this analysis. There was no significant difference in the overall autoreactivity at 1, 3, or 6 months after rituximab therapy (1-way paired-sample Wilcoxon’s test, *P* = 0.625 at 1 month, 0.125 at 3 months, and 0.3125 at 6 months) (Figure 2C). While PhIP-Seq enrichment does not report on absolute immunoglobulin levels, the levels of the disease-causing autoantibody (either anti-AChR or anti-MuSK antibodies) in myasthenia gravis were measured independently by a clinical radioimmunoassay (either Athena Diagnostic or Mayo Clinic Laboratory) in 6 of the 7 patients at the same time points. Although autoantibody levels minimally decreased in 3 patients, and moderately decreased in the other 3 patients following rituximab treatment, they never fell below the established positive cutoff for the assay (Supplemental Figure 3), suggesting that rituximab therapy was unable to quantitatively remove the pathogenic autoantibodies.

### CD19^+^ B cells are not required to maintain the autoreactome.

To evaluate the effect of CD19^+^ B cell depletion on the autoreactome, PhIP-Seq was performed on samples prior to, and approximately 6-months following, anti-CD19 CAR T cell therapy in 14 individuals being treated for lymphoma (CD19 CAR T demographics in Supplemental Table 1) who achieved and remained in remission, indicating successful depletion of the targeted cells. In 13 of the 14 patients, circulating CD19 cells were either persistently absent (defined as less than 10 CD19^+^ B cells per μL) or became absent following treatment. In the remaining patient, only a sample prior to therapy was available, and CD19 B cells were already absent, indicating that they likely remained absent following additional targeted CD19-depleting therapy (Supplemental Figure 4). None of the individuals had received an allogeneic hematopoietic cell transplant (HCT) in the preceding year (though 2 had received an autologous HCT), and 12 of the 14 patients were free of any additional B cell–depleting therapies for the 6 months prior to initial sample collection. IVIG is often administered to patients receiving B cell–depleting therapy, and 5 of the 14 patients received IVIG (CD19 sample clinical details in Supplemental Table 5).

**Figure 3:**
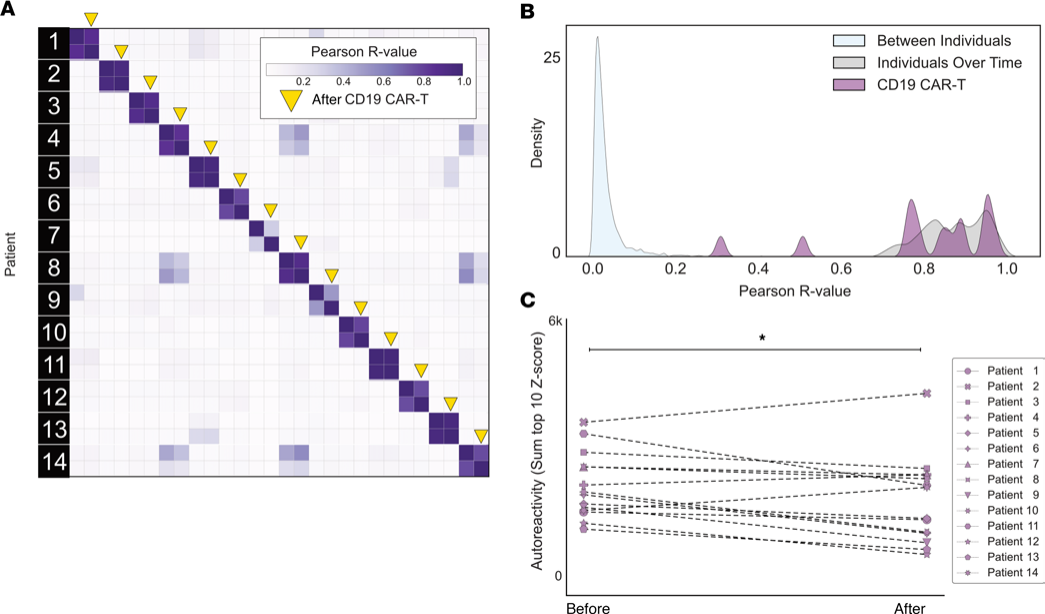
CD19 CAR T cell therapy has minimal effect on the autoreactome 6 months after treatment. (**A**) Correlation matrix showing Pearson’s *r* values of complete PhIP-Seq signal in 14 distinct individuals before and after anti-CD19 CAR T cell therapy. Yellow arrows represent the 6-month posttreatment time point. (**B**) Kernel density estimate plot showing distribution of correlation coefficients within each individual before and after therapy relative to the distribution among untreated individuals over time and between different individuals. (**C**) Line plots showing the autoreactivity (sum of top 10 PhIP-Seq Z scores relative to the 79 healthy controls) for each patient before and after treatment. One-sided paired-sample Wilcoxon’s test, *P* = 0.021.

The complete PhIP-Seq enrichment profile within each individual before and after CD19 CAR T cell therapy was once again compared. Despite depletion or persistently absent CD19 B cells in the setting of active CD19 CAR T cell therapy, the autoreactome remained remarkably stable over time (Pearson’s *r* coefficient median = 0.850; Q1 = 0.770, Q3 = 0.921) (Figure 3A). The overall distribution of correlation coefficients in the CD19 CAR T cell therapy group was not significantly different from intraindividual variation (Mann Whitney *U* *P* = 0.284) The distribution of correlation values in 12 of the 14 (85.7%) patients fell within the distribution (2 standard deviations from the mean) of longitudinal samples from healthy individuals who received no interventions (Figure 3B).

Using the same analysis approach as described for rituximab, we assessed differential PhIP-Seq enrichments using the sum of top 10 most-enriched protein targets before and after CD19 CAR T cell treatment (see Methods). Of the 14 individuals evaluated, 11 had a decrease in enrichment values while the remaining 3 had an increase, and levels overall were significantly decreased (1-sided paired-sample Wilcoxon’s test, *P* = 0.021) (Figure 3C). However, the size of the effect was minimal (median percentage decrease in autoreactivity of 11.9%). These results suggest that similar to rituximab, sufficient immunoglobulin producing cells remain after treatment such that the pattern of autoreactivity by PhIP-Seq remains largely unaltered.

To orthogonally validate the PhIP-Seq results, sera from 9 of these 14 patients were assayed using a previously described, multiplexed micro-bead assay consisting of 55 known protein autoantigens, each of which was covalently bound to microbeads with distinct bar codes (48). The list of autoantigens included proteins targeted in connective tissue diseases such as SLE, scleroderma, and myositis as well as secreted proteins such as cytokines, chemokines, and growth factors. Additionally, antibody signal to 21 viral antigens was tested to determine whether antibodies targeting both self-proteins and viral proteins respond similarly in the absence of CD19^+^ B cells (see Supplemental Table 6 for a list of antigens). Because IVIG contains autoantibodies that confound measurements in bead-based assays (our unpublished observations), we excluded all patients who had received IVIG within 8 weeks of the initial blood draw (before CAR T) or had received interim IVIG between the pre–CAR T cell and post–CAR T (6 months after) blood draw. Nine of our 14 patients met these stringent criteria and were included in these experiments.

As expected, a minority of autoantigens were recognized by serum IgG autoantibodies, including 3 intracellular proteins (thyroid peroxidase [TPO]; bactericidal permeability inducing protein [BPI]; and pyruvate dehydrogenase complex [PDC]) and 13 secreted proteins. Although levels of 14 of these 16 autoantibodies had decreased signal overall (sum of normalized MFIs; see Methods) following anti-CD19 treatment, this decrease was only statistically significant in 1 case (Supplemental Figure 5A). Among the 17 antiviral antibodies with meaningful signal, 12 were lower following CD19 therapy, but none were statistically significant (Supplemental Figure 5B). These data, generated with an orthogonal platform using full-length proteins as targets confirms that the autoreactome as well as IgG responses to viruses, remain largely stable over time following anti-CD19 CAR T cell therapy.

### The autoreactome is profoundly altered following depletion of BCMA^+^ B cells.

While CD19 is known to be expressed on a subset of antibody-secreting plasma cells (29), BCMA is a marker expressed on all plasma cells (31), making it an attractive target for broad autoantibody depletion. To assess the effect of BCMA CAR T on the autoreactome, we performed PhIP-Seq on serum samples from 9 individuals before, and approximately 6 months following, successful treatment with BCMA-targeted CAR T cell therapy (BCMA demographics in Supplemental Table 1). All 9 individuals had confirmed depletion of plasma cells in bone marrow following anti-BCMA CAR T cell treatment (Supplemental Figure 6). Each individual was being treated for multiple myeloma, none had received a HCT in the previous year, and 6 of the 9 had not received any additional B cell–depleting therapy in the prior year. All posttreatment samples were collected at least 56 days from the last dose of IVIG, and 3 of the 9 patients never received interim IVIG (BCMA sample clinical details in Supplemental Table 5).

**Figure 4:**
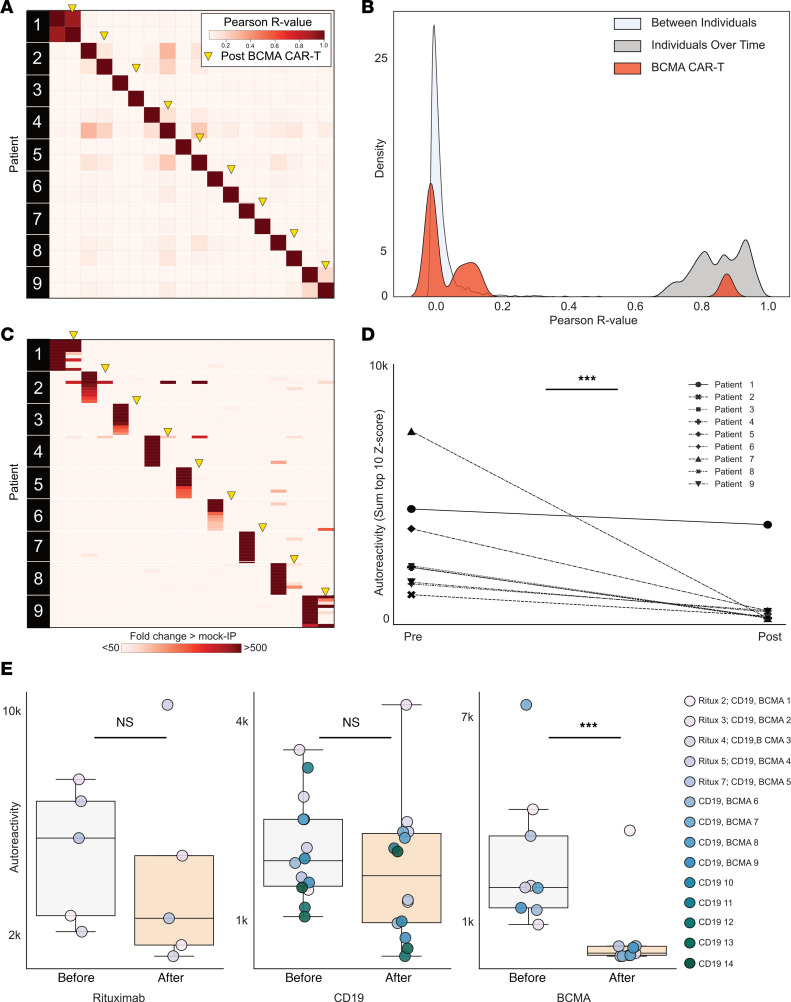
Anti-BCMA CAR T cell therapy significantly alters the autoreactome. (**A**) Correlation matrix showing Pearson’s *r* values of complete PhIP-Seq signal in 9 distinct individuals before and after anti-BCMA CAR T cell therapy. Yellow arrows represent the 6-month posttreatment time point. (**B**) Kernel density estimate plot showing distribution of correlation coefficients within each individual before and after BCMA-targeted therapy relative to the distribution among untreated individuals over time and the distribution between different individuals. (**C**) Heatmap showing top 10 autoreactivities (sum of top 10 PhIP-Seq *Z* scores relative to the 79 individuals acting as healthy controls) in each individual before and after treatment. (**D**) Line plots showing the autoreactivity (sum of top 3–12 PhIP-Seq *Z* scores relative to the 79 healthy controls, therefore accounting for potential paraprotein confounding) for each patient before and after treatment. One-sided paired-sample Wilcoxon’s test, *P* = 0.0019. (**E**) Box plots showing the relative distributions of autoreactivity before and after treatment with rituximab (sum of top 10), anti-CD19 CAR T cell therapy (sum of top 10), and anti-BCMA CAR T cell therapy (sum of top 3 through 12 autoreactivities). Anti-BCMA CAR T cell treatment cohort, Mann-Whitney *U*, *P* = 0.42, with a median percentage decrease of 52.3%; anti-CD19 CAR T cell treatment cohort, Mann-Whitney *U*, *P* = 0.206, with a median percentage decrease of 11.9%; and anti-BCMA CAR T cell treatment cohort, Mann-Whitney *U*, *P* = 0.003, with a median percentage decrease of 95.6%. ***P < 0.005.

The complete PhIP-Seq enrichment profile obtained for each individual before and after anti-BCMA CAR T cell therapy was compared. In contrast to CD19- and CD20-targeting therapies, the autoreactome was essentially devoid of any similarity following BCMA-targeted therapy (Pearson’s *r* value median = 0.006; Q1 = 0.002, Q3 = 0.130) for 8 of the 9 individuals (Figure 4A). The autoreactome of 1 individual remained unaltered (*r* = 0.894). This individual was subsequently found to have relapsed around the time of sample acquisition, indicating potential failure of the CAR T cell treatment. The overall distribution of correlation coefficients in the BCMA CAR T cell therapy group was significantly different from alterations in healthy individuals over time without interventions (Mann Whitney *U*, *P* = 0.000012) (Figure 4B). The samples from the individual with disease relapse were the only set whose autoreactome remained within 2 standard deviations from the mean of individuals over time without treatment. Remarkably, of the remaining 8 individuals, 7 had autoreactome correlation values following anti-BCMA CAR T cell therapy that fell within 2 standard deviations of the mean of samples taken from entirely different individuals. The observed complete “reset” of the autoreactome in these patients suggest that successful treatment with anti-BCMA CAR T cells sufficiently removes a lifetime of accumulated antibody-producing plasma cells.

While the overall autoreactive profile in sera following anti-BCMA CAR T cell treatment is markedly altered, we also examined changes to the most differentially enriched protein targets from pretreatment samples. Patients with multiple myeloma have a monoclonal expansion of a single plasma cell that secretes paraprotein antibody. Because paraprotein is potentially autoreactive, and because it is known to dramatically decrease following BCMA CAR T cell treatment in multiple myeloma, we removed the top two enriched protein targets from each individual prior to analysis to minimize the chance that our results were being confounded by changes in paraprotein level. The autoreactivity levels for the top 3–12 proteins decreased in every patient follow anti-BCMA CAR T cell therapy, and the overall change was statistically significant (1-sided paired-sample Wilcoxon’s test, *P* = 0.0019) (Figure 4, C and D). Unlike anti-CD19 CAR T cell treatment, in which the size of the decrease was minimal, there was a 95.6% decrease in PhIP-Seq enrichment following anti-BCMA CAR T cell treatment (Figure 4E). To ensure these findings were similar if the top 10 protein targets were used (and potential confounding by paraprotein is not accounted for), the same analysis was performed without the removal of the top 2 autoreactivities, with similar findings (Supplemental Figure 7).

To orthogonally validate the PhIP-Seq results and further explore the effects of plasma cell depletion on antiviral antibodies, we used the same multiplexed bead-based arrays described earlier to characterize IgG binding to 55 autoantigens and 21 viral proteins. However, because IVIG is routinely given following BCMA CAR T cell therapy, only 3 of the original 9 patients were IVIG free, so samples from 4 additional patients meeting the stringent criteria outlined previously were used (demographics and sample details in Supplemental Tables 7 and 8).

Of the same 16 measurable autoantigens analyzed in the anti-CD19 CAR T cell treatment cohort, 13 had overall decreased IgG binding (sum of normalized MFIs; see Methods) following anti-BCMA CAR T cell treatment. In contrast to the CD19 CAR T cohort in which there was only 1 autoantibody with a statistically significant decrease, levels of 9 of the 16 measured autoantibodies significantly decreased following anti-BCMA CAR T cell therapy (Supplemental Figure 8A). In addition, unlike the CD19 CAR T cohort, in which levels of none of the antiviral antibodies significantly decreased, all 17 of the measured antiviral antibodies decreased following BCMA therapy, and 8 of these decreases were statistically significant (Supplemental Figure 8B).

## Discussion

Adaptive immune responses targeting self rather than foreign proteins are a hallmark of autoimmune disease. Previous work has noted the existence of circulating self-reactive antibodies in healthy individuals. This work mostly focused on “natural autoantibodies,” which are thought to arise without antigen stimulation and to consist primarily of unmutated, polyreactive, often IgM-class antibodies (34, 35). Despite considerable interest, the role of these antibodies in health and disease remains unclear, though there is evidence of variable and context-dependent roles; the autoantibodies can contribute to, or protect against, inflammatory and autoimmune disease (36, 49–58). The bulk of previous work describing natural autoantibody repertoires has focused on the determination of shared autoantibody subsets among individuals. Recent work employing larger protein arrays identified a small subset (77 common autoantibody targets) that was frequently shared among both diseased and healthy individuals (37). Instead of focusing on a small subset of shared antigens, we evaluated the comprehensive set of possible peptide autoreactivities spanning the entire proteome, revealing highly individual, longitudinally stable profiles. The overwhelming uniqueness of individual profiles, rather than the small similarities, separates this work from prior investigations and allowed for an unbiased and broad evaluation of immune modulating therapies.

It has also been previously shown, using a small number (~200) of antigen targets chosen for autoimmune disease relevance, that individuals have distinctive patterns of autoantibodies that persist for up to a year (39). Here, we substantially expanded on this notion using a proteome-wide platform and found autoreactive profile stability exceeds 7 years and is not defined only by disease related antigens. Similar to Neiman et al., we found the unique nature of individual profiles may represent “serological fingerprints” or bar codes, and the approximately 2,000 times larger library used here may allow for more accurate identification of individuals, including forensic applications (39).

By defining and quantifying the autoreactome, we introduce what we believe to be a new tool for evaluating the relative efficacy of various immunomodulatory treatments in altering autoantibody profiles. This, in turn, yields insights into the cellular origins of the autoantibodies contained within the autoreactome. During a humoral immune response, plasma cells of varied lifespans are generated, with short-lived plasmablasts providing rapid antibody production in the acute phase and long-lived plasma cells sustaining durable antibody titers. While foreign antigen-specific long-lived plasma cells are known to persist for decades in humans (43, 59, 60), the cellular phenotype of pathogenic plasma cells targeting autoantigens remains controversial. Because individual autoantibodies decline following therapeutic B cell ablation with rituximab or anti-CD19 CAR T cell therapy, despite a lack of CD19 and CD20 expression on the majority of plasma cells (27, 61, 62), the prevailing model holds that the continuous generation of short-lived plasma cells from memory B cell precursors underlies the autoantibody repertoire (63). In contrast with this view, we show that not only is the broader intraindividual autoreactome stable over time, but that this autoantibody repertoire is resistant to therapeutic B cell ablation with rituximab (anti-CD20) and anti-CD19 CAR T cells. Given the expanded options for targeting B cells and plasma cells in systemic autoimmunity, this enhanced understanding of autoreactome biology has important clinical implications. Indeed, by using the autoreactome as a proxy for the autoantibody repertoire within an individual, we showed here that depletion of plasma cells via anti-BCMA CAR T cell therapy drastically alters the autoantibody repertoires. In most cases, the autoreactome of a sample after treatment with BCMA CAR T cells appeared as similar to an entirely different person as to themselves prior to treatment. In addition to the important clinical implications, this finding definitively confirms that the PhIP-Seq detected autoreactome we describe in this paper is indeed measuring autoantibodies produced by plasma cells.

It has been shown that rituximab reduces the level of certain autoantibodies (42) and has clinical efficacy in many autoimmune disease settings, which may relate more to the antigen presentation function of CD20^+^ B cells, as proposed by others (64). Additionally, CD19 CAR T cell therapy has recently been shown to be effective in the treatment of refractory SLE (27) and other autoimmune diseases (65) and to decrease the levels of certain autoantibodies, including anti–double-stranded DNA (dsDNA) antibodies. From this lens, our finding that the autoreactome was minimally affected by CD19 CAR T cell therapy suggests either that CD19^+^ B cells may have pathological effector functions beyond autoantibody production or that pathologic autoantibodies in autoimmune disease states are more likely to be derived from CD19^+^ B cells. In addition, the autoreactome we described here exclusively looks for autoantibodies targeting the human proteome and therefore does not detect potentially important additional types of autoantibodies, such as those targeting DNA (anti-dsDNA). Because both anti-CD19 CAR T and anti-BCMA CAR T are primarily used in the treatment of hematologic malignancy, our cohorts received chemotherapy prior to CAR T cell therapy and, therefore, had low CD19 and plasma cells numbers, even prior to the initiation of therapy. Future studies will be required to further understand the relationship of the pre– and post–CAR T cell treatment autoreactomes, and their relationship to clinical efficacy, in the specific context of autoimmune diseases.

Our broad-based approach to screen autoantibodies at a proteome-wide level allows a more comprehensive and unbiased assessment of the effect of therapies on the specificity of the autoreactome. We posit that this approach could greatly complement emerging data on the use of CAR T cell therapies that are being utilized against severe autoimmune diseases. Indeed, since the introduction of BCMA-directed CAR T cell therapy, attempts have been made to measure attributable alterations in autoantibodies but have been limited by an inability to identify sufficient autoantibodies in pretreated samples in order to track treatment-associated changes longitudinally (43). Even attempts to measure changes in viral-antibody titers have been limited by confounding IVIG, preventing longitudinal assessments and limiting studies to single time point snapshots (44). Our results overcome these limitations and reveal the profound effects of BCMA-directed therapies on autoantibody repertoires relative to rituximab or CD19 CAR T.

The prevalence and burden of autoimmune disease continues to rise (66, 67), yet the precise mechanisms for the development of autoreactivity remain unclear. There is mounting evidence that combinations of genetic and environmental risk factors, including viral infections, predispose individuals to disease. In addition to the emerging therapies already discussed, other new therapies exist that can halt or prevent the progression of certain autoimmune diseases, including type 1 diabetes (68). By defining the autoreactome and describing the expected longitudinal changes over time, we introduce a tool that can be applied for additional basic and translational studies in autoimmunity. Future work will be needed to explore the autoreactome to determine at what age it develops and becomes stable, whether there are shared features of autoreactive epitopes, what drives the development of the autoreactivities, and what biological role these autoreactive antibodies may have in health and disease. Beyond the already discussed application for evaluating the relative effectiveness of various emerging therapies, there are additional potential future applications of this work, including tracking longitudinal autoreactome changes in individuals prior to, and following, the development of specific autoimmune diseases. This may allow for the identification of autoreactive signatures that precede the development of symptoms and, thereby, produce potential novel biomarkers to identify patients in whom autoimmune disease could be prevented.

## Methods

### Sex as a biological variable.

The conditions affecting the individuals included in this research occur in both males and females. Therefore, both male and female participants are included in this study.

### Patients.

The healthy control cohort consisted of plasma from 79 self-reported healthy individuals collected prior to the COVID-19 pandemic, which were obtained as deidentified samples from the New York Blood Center.

For the bead-based protein arrays, sera and/or plasma was used for internal controls and validations, as has been previously described, as deidentified samples (70). Negative controls included a pediatric sample from an infant between 6 and 12 months of age, which was a gift from the Bali Pulendran lab (Institute for Immunity, Transplantation and Infection, Stanford University School of Medicine, Stanford, CA, USA), as well as samples from 3 healthy individuals with known CMV-seronegative status confirmed by ELISA from the Stanford Blood Center. Positive controls include plasma samples derived from participants with autoimmune or other types of diseases with known reactivity patterns (e.g., TPO, PDC-E2, FGF7, proteinase 3, IL-11, CXCL-13), which were purchased from ImmunoVision or were obtained from Stanford Autoimmune Diseases Biobank and Oklahoma Medical Research Foundation (a gift of Judith James, Oklahoma Medical Research Foundation, Oklahoma City, Oklahoma, USA). A total of 12 positive control patient samples were used.

Longitudinal plasma samples were obtained prior to the COVID-19 pandemic from a set of 7 community dwelling, cognitively unimpaired, healthy older adults recruited through the Brain Aging Network for Cognitive Health at the UCSF. All participants were screened at baseline, and enrollment criteria excluded individuals with severe psychiatric illness, neurologic disorders (e.g., epilepsy, multiple sclerosis), and medical conditions that could affect cognition (e.g., recent substance use disorders, active chemotherapy). Following a comprehensive neurobehavioral evaluation, participants were classified cognitively normal per consensus conference with board-certified neurologists and neuropsychologists. Inclusion in the current study was contingent on no known autoimmune conditions at baseline or follow-up visits, either active or inactive at the time of plasma collection.

For the cohort of patients with myasthenia gravis (used to evaluate the effects of IVIG and rituximab), deidentified serum samples from patients with myasthenia gravis, with laboratory-confirmed AChR or MuSK autoantibody serostatus, were retrieved from a biorepository established at the Yale School of Medicine.

The CAR T cell cohorts consisted of adults aged ≥18 years with relapsed or refractory CD19^+^ or BCMA^+^ hematologic malignancies who received lymphodepleting chemotherapy with cyclophosphamide and fludarabine followed by anti-CD19 or anti-BCMA-CAR T cells at Fred Hutchinson Cancer Center. Sera and PBMCs were prospectively collected once prior to lymphodepleting chemotherapy and at approximately 6–12 months after CAR T cell infusion among individuals who achieved durable remissions and received no subsequent antitumor therapies.

### PhIP-Seq.

All PhIP-Seq was performed similar to our previously published multichannel protocol (17), with minor adjustments, as outlined in our new protocol: https://www.protocols.io/view/derisi-lab-phage-immunoprecipitation-sequencing-ph-czw7x7hn?step=14.1

As previously described (11), our human peptidome library consisted of a custom-designed phage library of 731,724 unique T7 bacteriophages, each presenting a different 49–amino acid peptide on its surface. Collectively, these peptides tile the entire human proteome, including all known isoforms (as of 2016) with 25 amino acid overlaps. One milliliter of phage library was incubated with 1 μL human serum overnight at 4°C and immunoprecipitated with 25 μL of 1:1 mixed protein A and protein G magnetic beads (Thermo Fisher Scientific, 10008D and 10009D). These beads were than washed, and the remaining phage-antibody complexes were eluted in 1 mL *E*. *coli* (BLT5403, EMD Millipore) at 0.5–0.7 OD and amplified by growth in an incubator at 37°C. This new phage library was then reincubated with the same individual’s serum, and the previously described protocol was repeated. DNA was then extracted from the final phage library, bar coded, and PCR amplified, and Illumina adaptors were added. Next-generation sequencing was then performed using an Illumina sequencer to a read depth of approximately 1 million per sample.

### Bead-based protein array.

A 76-plex protein array comprising a 55-plex autoantigen protein subarray and a 21-plex viral protein subarray was constructed as previously described (48), with modifications. In brief, antigens (Supplemental Table 6) were conjugated to uniquely bar-coded carboxylated magnetic beads (MagPlex-C, Luminex Corp). The beads were stored in aliquots at –80°C after conjugation and thawed on the day of the experiment. 45 μL diluted serum or plasma sample (1:100 in PBS 1% BSA) was transferred into the 384-well plate (Greiner BioOne) containing 5 μL bead array per well. Samples were incubated for 60 minutes on a shaker at room temperature and then at 4°C overnight. The following day, beads were washed 4 times with 60 μL PBS-Tween on a plate washer (EL406, Biotek) and then incubated with 50 μL 1:1,000 diluted R-phycoerythrin–conjugated Fc-γ–specific goat anti-human IgG F(ab′)2 fragment (Jackson ImmunoResearch, 109-116-170) for 30 minutes. The plate was washed 4 times with 60 μL PBS-Tween and resuspended in 50 μL PBS-Tween prior to analysis using a FlexMap3D instrument (Luminex Corp.) and measuring median fluorescence intensity (MFI) with at least 50 beads per bar code for each sample. Most of the individual antigens that had been conjugated to beads were qualified prior to mixing using antibodies directed against epitope tags, monoclonal antibodies specific for the antigen, or prototype human plasma samples derived from participants with autoimmune diseases with known reactivity patterns (e.g., Scl-70^+^ systemic sclerosis sera; SSA^+^, which is associated with lupus and Sjögren’s syndrome; ribonucleoprotein^+^ [RNP^+^] sera, which are associated with lupus and mixed connective tissue disease; and APS1 sera, which are reactive with multiple cytokines; data not shown). Binding events were displayed as MFI. MFI values were normalized by subtracting bare-bead MFI values. Replicate MFI values were averaged.

### Enumeration of B cells and plasma cells.

B cells were quantified using a research flow cytometry panel (representative gates shown in Supplemental Figure 9). Briefly, PBMCs were incubated on ice for 30 minutes with fluorescently labeled antibodies (in fluorescence-activated single-cell sorting buffer containing Dulbecco’s phosphate-buffered saline and 1% newborn calf serum [Life Technologies]) before its analysis on a FACSymphony A5 (BD Bioscience) flow cytometer. The following antibodies were included for cell labeling: fixable viability stain 700 (BD, catalog 564997), anti-CD19 BV421 (HIB19, BD, catalog 562440), anti-CD45 BV510 (HI30, BD, catalog 563204), anti-CD3 BV605 (UCHT1, BioLegend, catalog 300460), anti-CD16 BV711 (3G8, BD, catalog 563127), and anti-CD14 BV711 (M0P-9, BD, catalog 563372). FlowJo software version 10.7.1 was used for analyses, with flow cytometry proportions multiplied by absolute lymphocyte counts to calculate total CD19^+^ B cell numbers. Plasma cells in the bone marrow were quantified based on detection of CD138^+^ plasma cells in clinically obtained bone marrow core biopsies by immunohistochemistry in the University of Washington Hematopathology Laboratory.

### Analysis of PhIP-Seq.

As previously described (11), next-generation sequencing reads from fastq files were aligned at the level of amino acids using RAPSearch2. All results represent the average of technical replicates, except for the 79 individuals acting as healthy controls, from whom only 48 samples from 24 of the individuals were used to perform in technical replication. All human peptidome analysis was performed at the gene level, in which all reads for all peptides mapping to the same gene were summed, and 0.5 reads were added to each gene to allow inclusion of genes with 0 reads in mathematical analyses. Within each individual sample, reads were normalized by converting to the percentage of total reads. To normalize each sample against background nonspecific binding, a FC over mock IP was calculated by dividing the sample read percentage for each gene by the mean read percentage of the same gene for the AG bead only controls. This FC signal was then used for side-by-side comparison between samples and cohorts. FC values were also used to calculate *Z* scores for specific cohorts relative to defined controls. For the gene ontology analysis identifying cellular locations of autoantigens, PANTHER (69) was used categorize genes into a Panther GO Slim Cellular Component.

### Analysis of multiplexed bead arrays.

Antibody reactivity to each target for each sample was measured in MFI. Data are only shown for targets that have an MFI of at least 1,000 MFI units at any time point for any patient. To adjust for background binding to beads only, each sample was additionally run on unconjugated beads, and bare bead MFI was subtracted to create an adjusted MFI for each target antigen within the same corresponding sample. To account for nonspecific binding from human serum, a sample from an infant control known to lack antibodies to any of the viral or human targets was run in duplicate. The mean of the adjusted MFIs for the control duplicates was calculated for each target and further subtracted from each corresponding target from each sample. A normalization function was then performed such that all sample values for each target antigen ranged from 0 to 1, referred to as “normalized MFI.”

### Statistics.

All statistical analyses were performed in Python using the Scipy Stats package. To calculate the overall similarity of PhIP-Seq–detected autoreactive profiles among different samples, a Pearson’s correlation coefficient was calculated using all FC over mock IP values (unless specifically stated otherwise) for each autoantigen in each sample relative to each autoantigen in each other sample. For comparisons of distributions of PhIP-Seq signal (either autoreactivity scores or Pearson’s *r* values) between two groups, a nonparametric Mann-Whitney *U* test was utilized. To determine whether specific interventions (IVIG, rituximab, anti-CD19 CAR T cell, or anti-BCMA CAR T cell) directionally decreased PhIP-Seq-detected autoreactivities, first an “autoreactivity” score was calculated by summing the top 10 autoantigen *Z* scores (relative to the 79 individuals acting as healthy controls) in each pretreatment sample. The sum of the *Z* scores from these same 10 autoantigens was again calculated following the intervention for each posttreatment sample from the same individual. These autoreactivity scores from before and after a treatment were paired for each individual and used as input for a 1-sided paired-sample Wilcoxon’s test. To determine whether anti-CD19 CAR T cell treatment or anti-BCMA CAR T cell treatment altered Luminex-detected antibody levels from the bead-based protein arrays, the pretreatment and posttreatment normalized MFI values were compared using a 2-sided paired-sample Wilcoxon’s test for each target antigen. *P* values of less than 0.05 were considered significant.

### Study approval.

Healthy control samples from the New York Blood Center were collected using retention tubes at the time of blood donations from volunteer donors who provided informed consent for their samples to be used for research. For the longitudinal healthy cohort, study procedures were approved by the UCSF Committee on Human Research, and all participants provided written informed consent (IRB #10-02076). For the bead-based assay, all 16 of the control patients (4 negative controls and 12 positive controls) provided informed consent for their samples to be used for research. The myasthenia gravis cohort sample was part of the Myasthenia Gravis Clinical Trial (EXPLORE-MG Registry, NCT03792659) and was obtained under the approval of Yale University’s Institutional Review Board. All patient samples from the CAR T cell treatment cohorts were obtained as part of a study approved by the Fred Hutchinson Cancer Center institutional review board (protocol 10080), and all participants provided informed consent in accordance with the Declaration of Helsinki.

### Data availability.

All PhIP-Seq data are publicly available via a Dryad digital repository (https://datadryad.org/stash/dataset/doi:10.5061/dryad.w3r2280z6). All supporting data values associated with the manuscript and supplemental materials are available in the Supporting Data Values file.

## Author contributions

AB, HL, MRW, JAH, SWJ, MSA, and JLD conceptualized the study. AB, JA, AFK, HK, ER, CYW, AS, PJU, AR, MK, MSA, and JLD provided methodology. AB, DJLY, AR, MK, JB, CMB, and BO performed or contributed to experiments. AB and JLD provided formal analysis. KZ, KD, VED, EQG, BC, DJG, JG, CJT, JAH, GM, RJW, and KCO acquired patient samples and clinical data. KZ, KD, KBC, JHK, VED, EQG, BC, DJG, JG, CJT, JAH, GM, KCO, and RS curated clinical data. AB and JLD wrote the original draft of the manuscript. AB, KCO, PJU, JAH, SWJ, MSA, and JLD reviewed and edited the manuscript. PJU, JAH, SWJ, MSA, and JLD supervised the study.

## Supplementary Material

Supplemental data

Supporting data values

## Version 1 05/16/2024

In-Press Preview

## Version 2 07/01/2024

Electronic publication
