# Supplementary material for: Unveiling the proteome-wide autoreactome enables enhanced evaluation of emerging CAR T cell therapies in autoimmunity
Source: J Clin Invest. 2024 May 16;134(13):e180012. doi: 10.1172/JCI180012 (PMC11213466; doi:10.1172/JCI180012)
Supplement: Supplemental data [file jci-134-180012-s005.pdf]

Supplemental Material

Supplemental Figures:

Supplemental Figure 1. Distribution of autoantibodies across healthy individuals

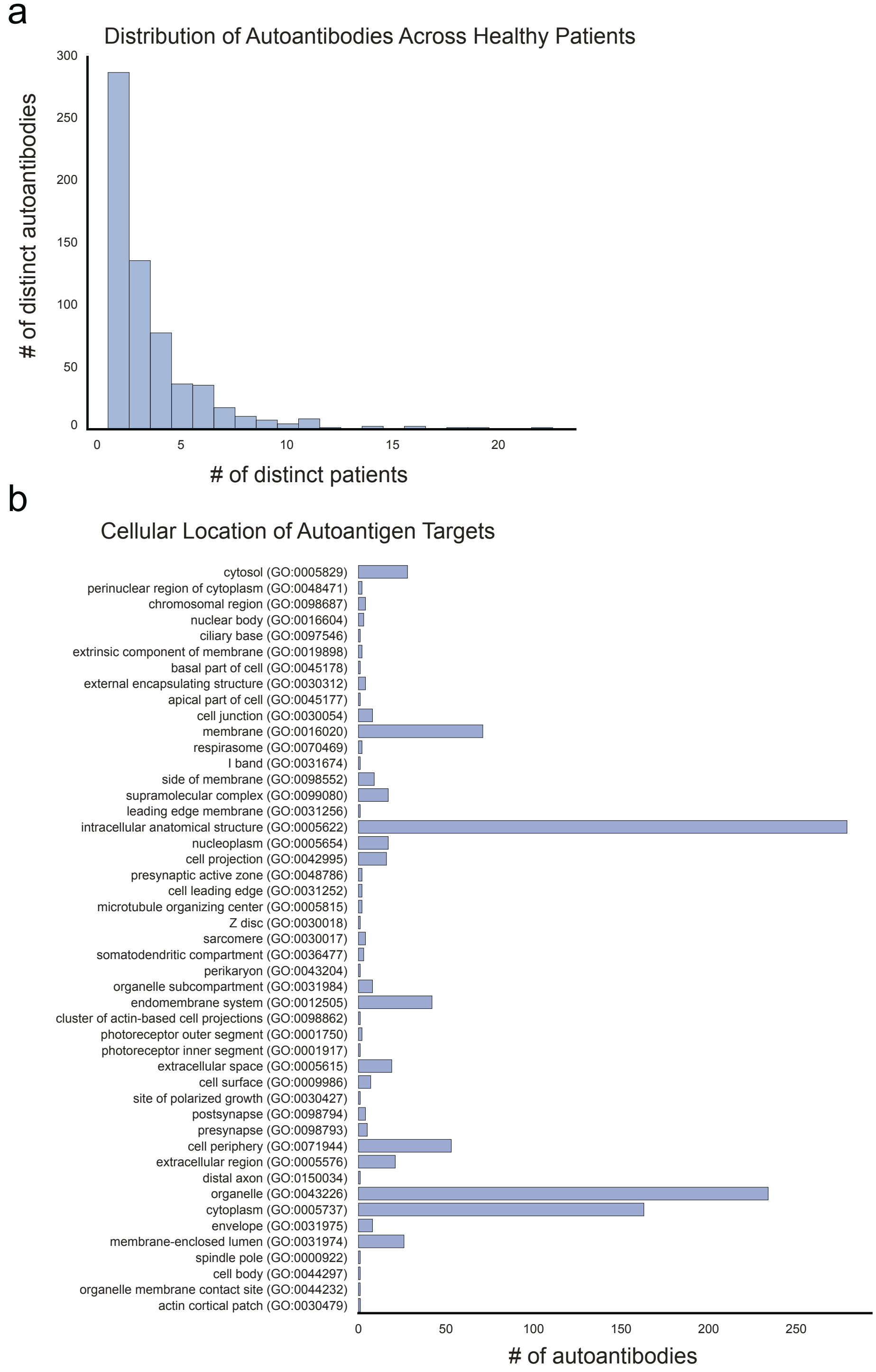

(a) Histogram showing the distribution of highly enriched autoantibodies within any one individual (for each of the 79 healthy individuals in the cohort, the top 10 autoantibodies measured by fold-change over mock-IP were identified, which yielded 623 unique autoantibody targets) across the entire cohort of 79 healthy individuals. **(b)** Barplot showing the cellular location (cellular anatomical entity (GO:0110165) determined by Panther GO Slim Cellular Component analysis mapped 570 of the 623 total autoantigens) of autoantibody targets.

Supplemental Figure 2. Administration of IVIG minimally alters the autoreactome

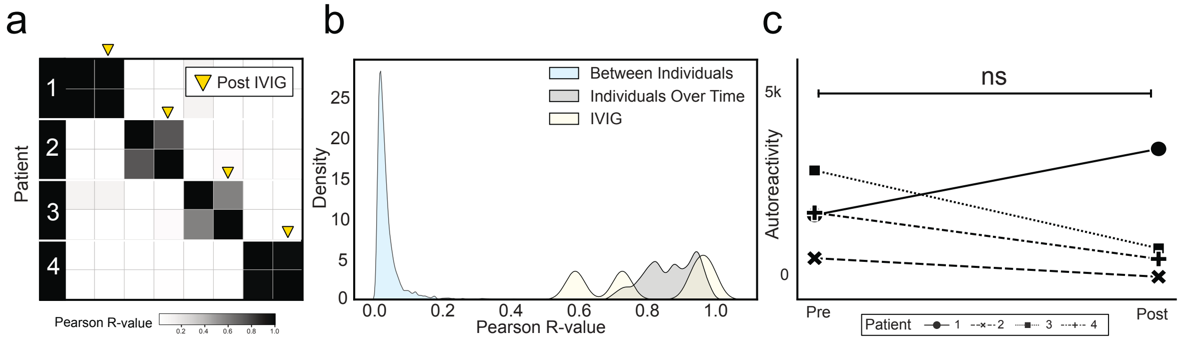

(a) Correlation matrices showing Pearson correlation coefficients of complete PhIP-Seq signal before and after IVIG administration. **(b)** Kernel density estimate plot showing distribution of Pearson R correlation coefficients from before and after IVIG administration relative to longitudinal samples from individuals over time, and between different individuals. **(c)** Lineplots showing the autoreactivity (sum of top 10 PhIP-Seq Z-scores relative to the 79 healthy controls) for each patient before and after IVIG administration. Paired-samples Wilcoxon test p-value 0.625.

Supplemental Figure 3. Changes in disease-causing autoantibody levels following rituximab

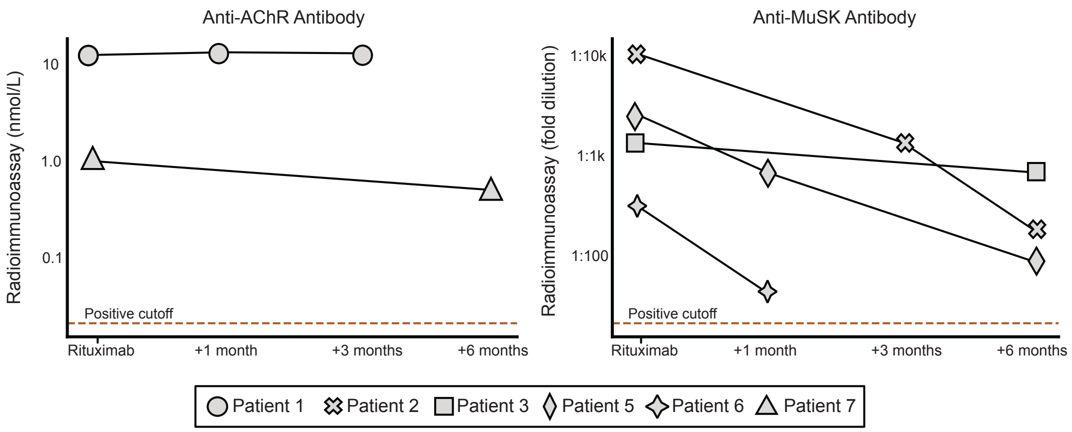

Left: Anti-AChR autoantibody levels measured by radioimmunoassay (RIA; Mayo Clinic Laboratory, unit=nmol/L) in 2 patients at 1 month, 3 months, and 6 months post-rituximab. RIA positive cutoff 0.02 nmol/L. **Right**: Anti-MuSK autoantibody titers measured by RIA (Athenia Diagnostics, unit=fold dilution) in a different 4 patients at 1 month, 3 months, and 6 months post-rituximab. Positive cutoff for is a fold dilution of greater than 1:20.

Supplemental Figure 4: CD19 cell counts following anti-CD19 CAR-T treatment

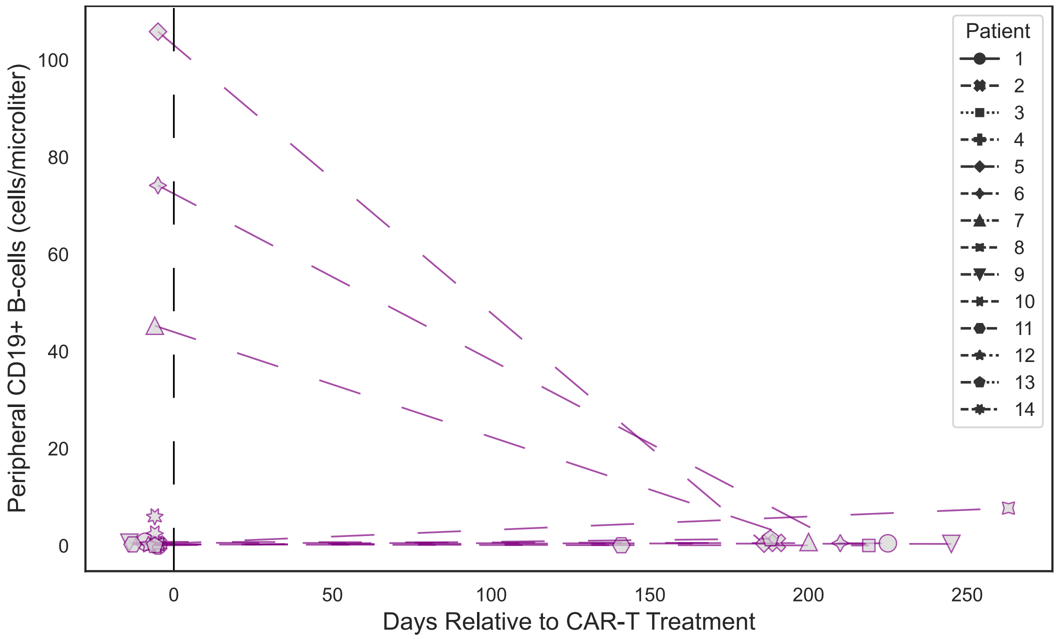

Lineplots showing the levels of CD19^{+} B cells in peripheral blood measured by flow cytometry at various timepoints relative to anti-CD19 CAR-T treatment, in each patient.

Supplemental Figure 5: Changes in multiplex bead-based protein array detected antibody levels following CD19 therapy

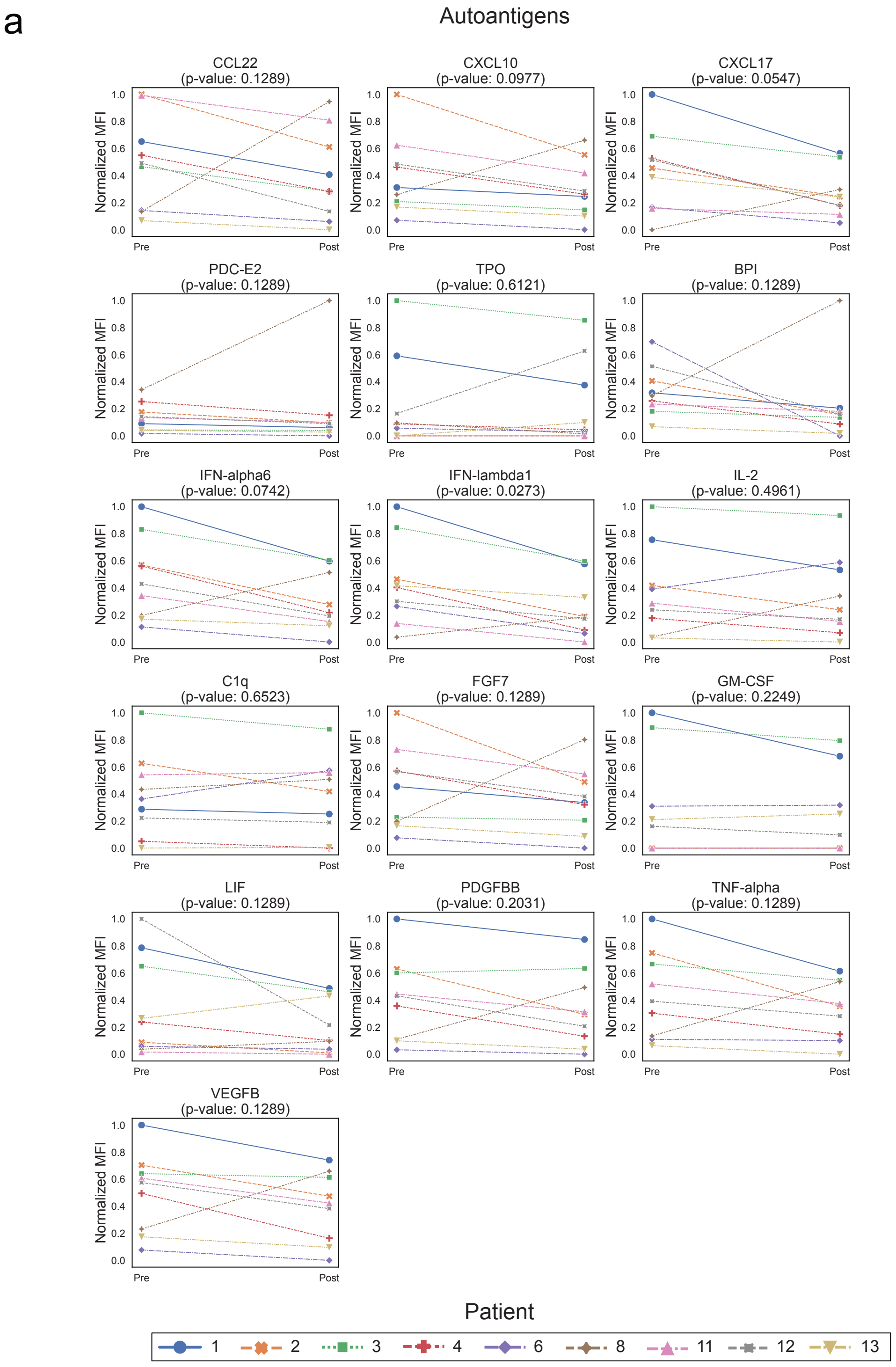

b

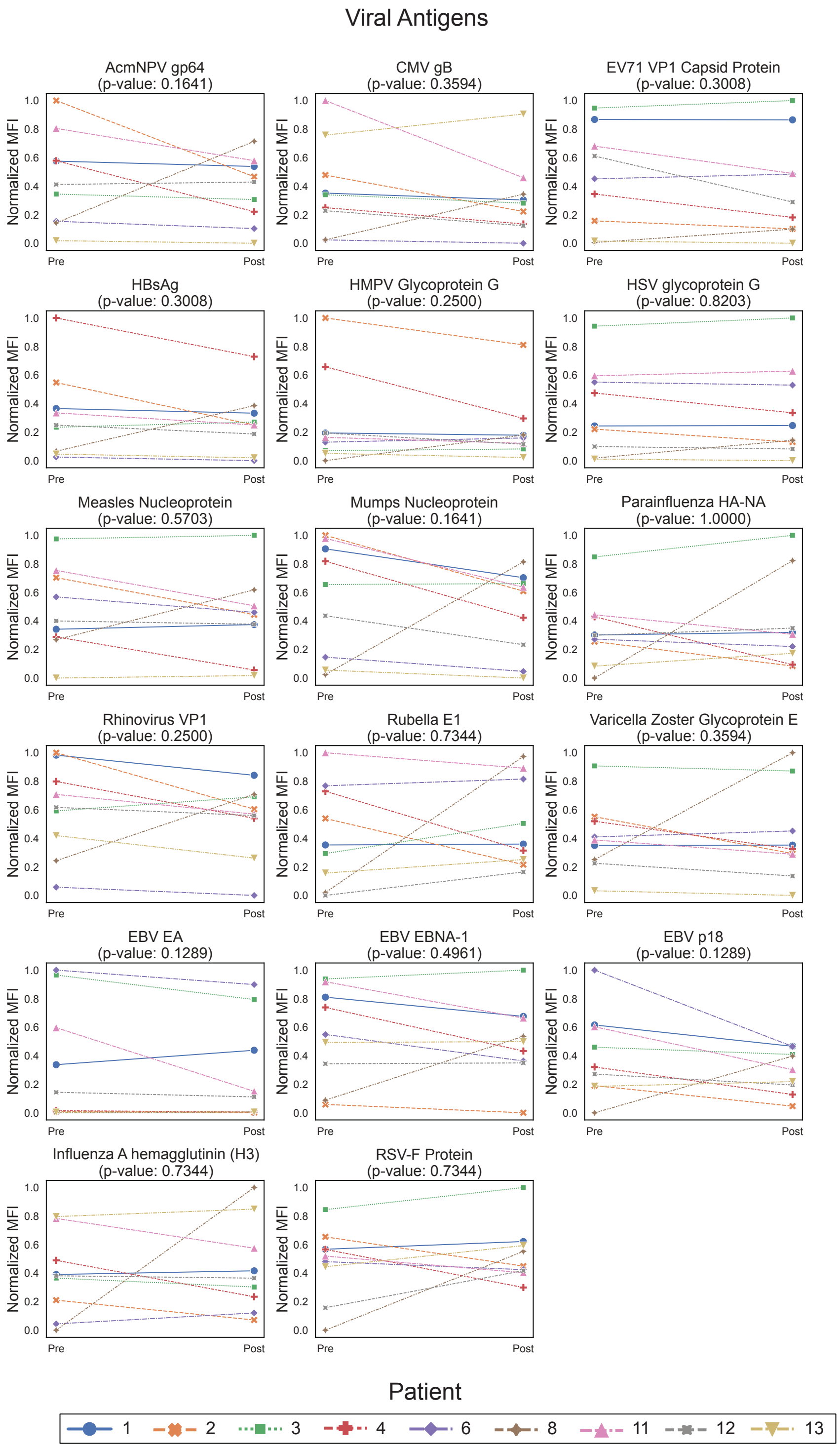

(a) Multiplex bead-based protein array detected normalized MFI values (see “Methods”) for each

of the 16 autoantigens with meaningful signal (see “Methods”) in each of the patients before and approximately 6 months after treatment with anti-CD19 CAR-T cells. P-values shown are from a paired-samples Wilcoxon test. **(b)** Multiplex bead-based protein array detected normalized MFI values (see “Methods”) for each of the 17 viral antigens with meaningful signal (see “Methods”) in each of the patients before and after treatment with anti-CD19 CAR-T cells. P-values shown are from a paired-samples Wilcoxon test.

Supplemental Figure 6: Changes in bone marrow plasma cell percentage following anti-BCMA CAR-T cell therapy

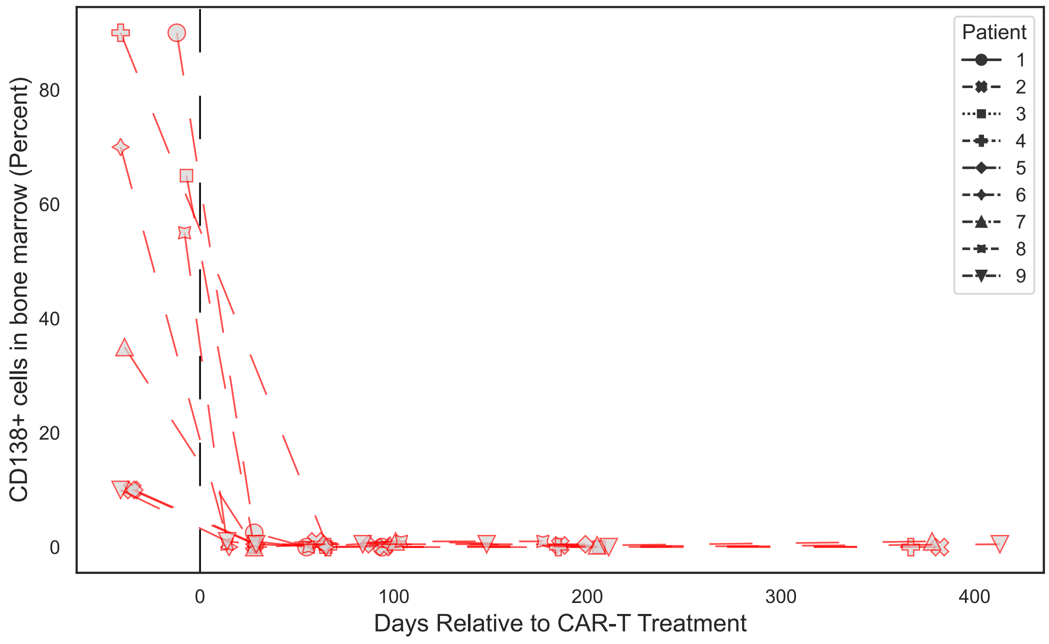

Lineplots showing the levels of plasma cells in the bone marrow measured by flow cytometry at various timepoints relative to anti-BCMA CAR-T treatment, in each patient.

Supplemental Figure 7: Changes in PhIP-Seq detected autoreactivities following anti-BCMA CAR-T cell therapy without controlling for potential confounding by paraprotein

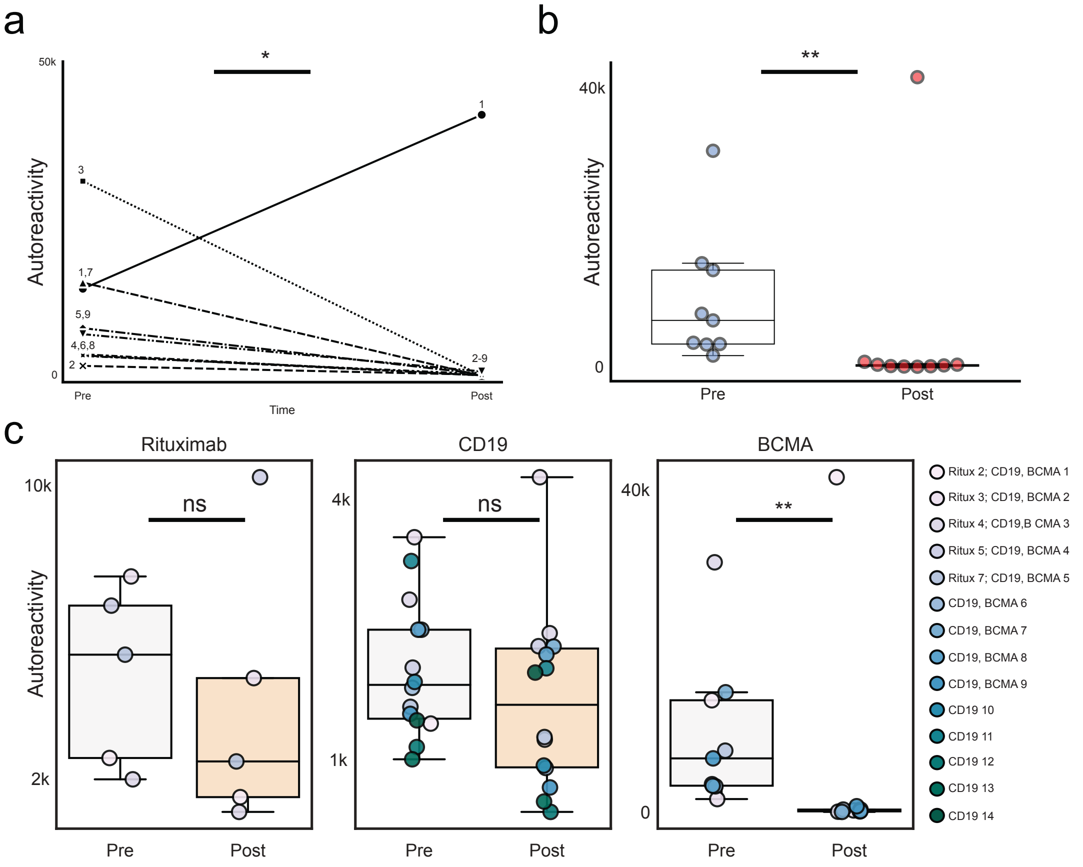

(a) Lineplots showing the autoreactivity (sum of top 10 PhIP-Seq Z-scores relative to the 79 healthy controls) for each patient before and after treatment. One-sided paired samples Wilcoxon test p-value=0.049. **(b)** Swarmplots showing the relative distributions of autoreactivity (sum of top 10 PhIP-Seq Z-scores relative to the 79 healthy controls) before and after treatment with anti-BCMA CAR-T. Mann-Whitney U p-value=0.006. **(c)** Boxplots showing the relative distributions of autoreactivity (sum of top 10 PhIP-Seq Z-scores relative to the 79 healthy controls) before and after treatment with rituximab, anti-CD19 CAR-T, and anti-BCMA CAR-T. rituximab treatment cohort Mann-Whitney U p-value=0.42 with a median percent decrease of 52.3%; anti-CD19 CAR-T treatment cohort Mann-Whitney U p-value=0.206 with a median percent decrease of

11.9%; anti-BCMA CAR-T treatment cohort Mann-Whitney U p-value=0.006 with a median percent decrease of 97.2%.

Supplemental Figure 8: Changes in multiplex bead-based protein array detected antibody levels following BCMA therapy

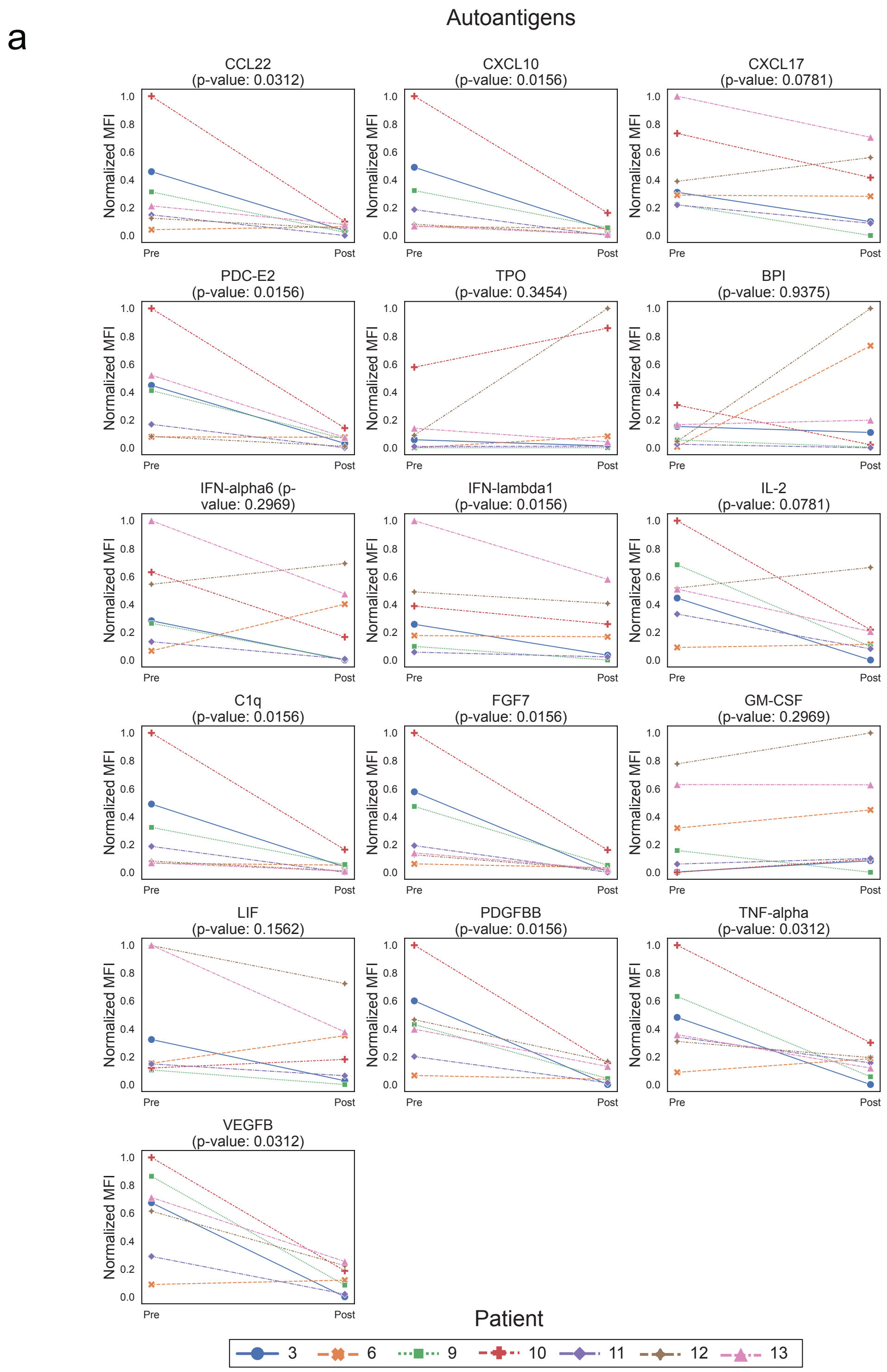

b

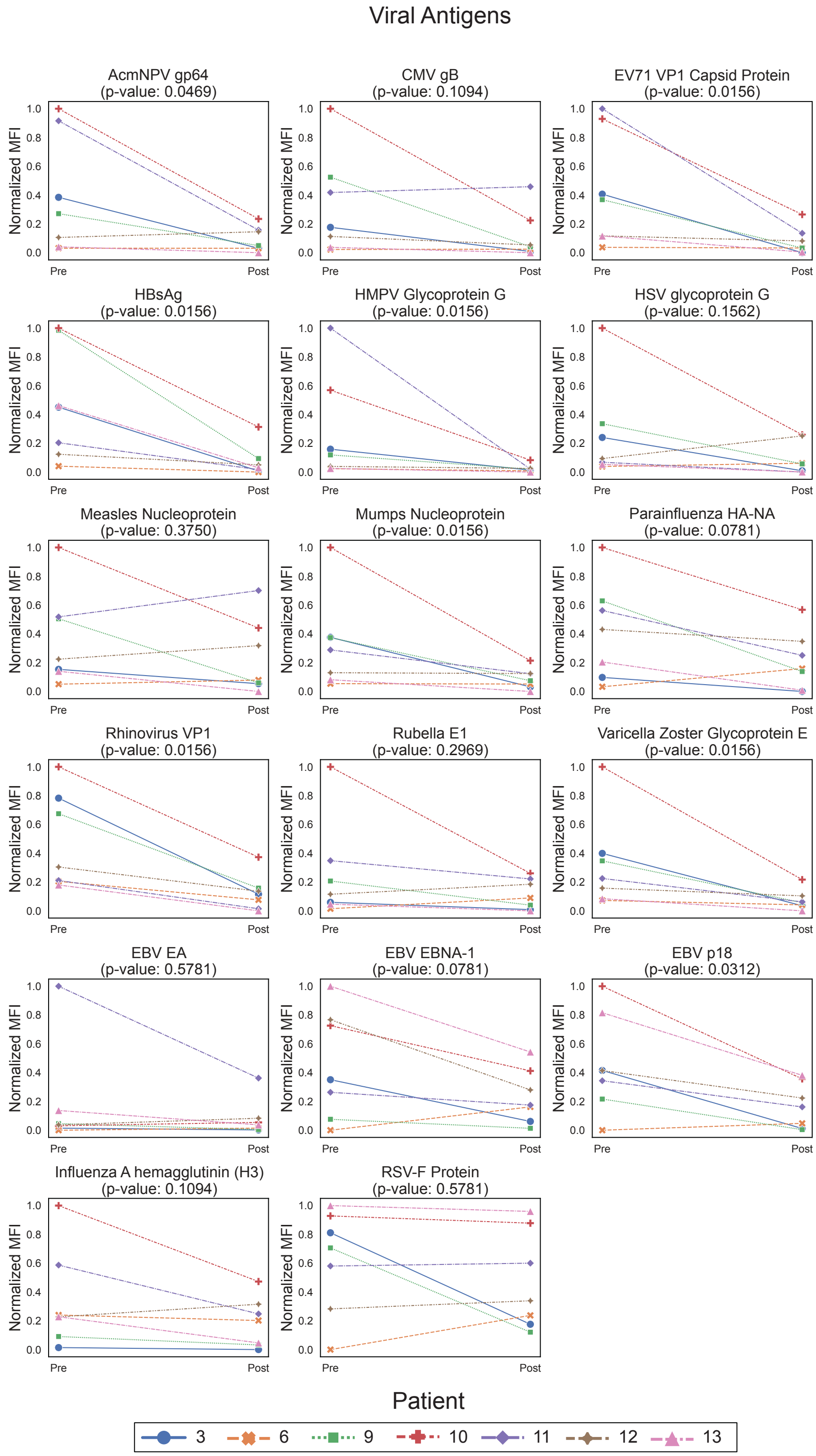

(a) Multiplex bead-based protein array detected normalized MFI values (see "Methods") for each

of the 16 autoantigens with meaningful signal (see “Methods”) in each of the patients before and after treatment with anti-BCMA CAR-T cells. P-values shown are from a paired-samples Wilcoxon test. **(b)** Multiplex bead-based protein array detected normalized MFI values (see “Methods”) for each of the 17 viral antigens with meaningful signal (see “Methods”) in each of the patients before and after treatment with anti-BCMA CAR-T cells. P-values shown are from a paired-samples Wilcoxon test.

Supplemental Figure 9: Flow cytometry gating strategy for identification of CD19^{+} B cells

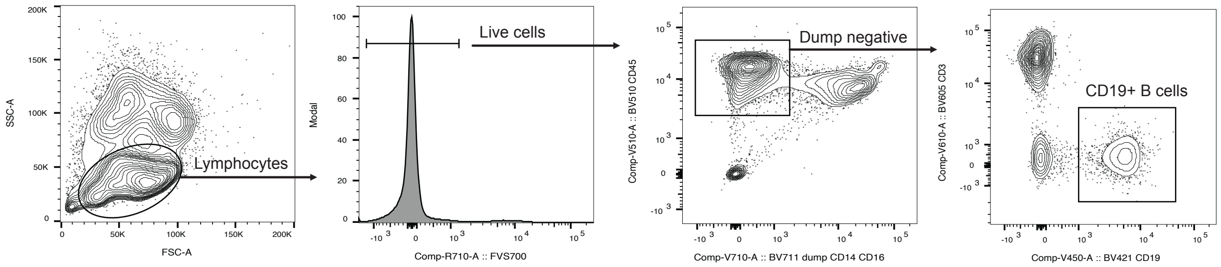

Representative flow cytometry gating for the identification and enumeration of CD19^{+} B-cells.

Supplemental Tables:

Supplemental Table 1: PhIP-Seq Cohort Demographics

	Healthy	Longitudinal	IVIG	Rituximab	CD19 CAR-T	BCMA CAR-T
**Number**	79	7	4	7	14	9
**Male sex (%)**	41 (51.9)	2 (28.6)	1 (25)	2 (28.6)	9(64.3)	4 (44.4)
**Median age in years (IQR)**	48 (36-55.5)	74 (71.5-76.5)	Ranges*	Ranges**	58 (47-64)	65 (60-70)

Race and Ethnicity (%)						
White	63 (77.7)	7 (100)	3 (75)	6 (85.7)	14 (100)	8 (88.9)
Black	1 (1.2)	0	0	1 (14.3)	0	0
Hispanic/Latinx	10 (12.3)	0	0	0	0	0
Asian/Pacific Islander	3 (3.7)	0	1 (25)	0	0	0
Multiple Races	3 (3.7)	0	0	0	0	0
Unknown/Other	1 (1.2)	0	0	0	0	1 (11.1)

* Age ranges at time of first sample collection: 26-30 years, n=1; 31-35 years, n=1; 35-40 years, n=1; 51-55 years, n=1.

**Age ranges at time of first sample collection: <20 year old, n=1; 21-25 years, n=1; 35-40 years, n=1; 61-65 years, n=2, 66-70 years, n=2.

Supplemental Table 2: Healthy Longitudinal Patient Clinical Details

ID	Duration	Autoimmunity	Cancer	Immunomodulation
Patient 1	63 months	None	None	None
Patient 2	25 months	None	Basal cell carcinoma	None
Patient 3	75 months	None	None	None
Patient 4	92 months	None	None	None
Patient 5	61 months	Sjögren’s Disease	None	None
Patient 6	50 months	None	None	None
Patient 7	77 months	None	None	None

Supplemental Table 3: IVIG Sample Details

ID	Sample Timing	Immunomodulation	Last IVIG Dose
**Patient 1**			
	Initial	None	Naïve
	6 weeks later	Prednisone/Imuran	3 weeks prior
**Patient 2**			
	Initial	None	Naïve
	14 weeks later	Prednisone	1 day prior
**Patient 3**			
	Initial	Prednisone	Naïve
	7 weeks later	Prednisone	2 days prior
**Patient 4**			
	Initial	None	Naïve
	21 weeks later	Prednisone	5 weeks prior

Supplemental Table 4: Rituximab Sample Details

ID	Autoantibody	Sample	Immunomodulation	Rituximab
**Patient 1**	**AchR**			
		1	Prednisone	Naïve
		2	Prednisone	1.1
		3	Prednisone	3.9
		4	Prednisone	3.4

		5	Prednisone	6.1
		6	Prednisone	24.6
**Patient 2**	**MuSK**			
		1	Prednisone	Naïve
		2	Prednisone	2.3
		3	Prednisone	5.1
		4	Prednisone	4.8
**Patient 3**	**MuSK**			
		1	Prednisone	6.1
		2	Prednisone	5.1
		3	None	68.9
**Patient 4**	**MuSK**			
		1	None	8
		2	None	7.9
		3	None	24.7
**Patient 5**	**MuSK**			
		1	None	17.8
		2	None	1.3
		3	None	8.6
		4	None	13.9
		5	None	18.8
		6	None	21
		7	None	5.7

		8	None	1.6
**Patient 6**	**MuSK**			
		1	Prednisone	Naïve
		2	Prednisone, Imuran	1.7
		3	Prednisone, Imuran	4.6
		4	Prednisone, Imuran	4.7
		5	Prednisone, Imuran	8.5
**Patient 7**	**AChR**			
		1	Prednisone, Imuran	5.7
		2	Prednisone, Imuran	7
		3	None	24.1

Supplemental Table 5. CAR-T Sample Details

CAR-T	Patient	Malignancy	Post-CAR-T Sample Timing (weeks)	Prior therapy (months pre-CAR-T)	Concurrent treatment (days prior to second sample)
**BCMA**					
	Patient 1	MM	51	IVIG, 6	IVIG, 117
	Patient 2	MM	27	IVIG, 4	IVIG, 56
	Patient 3	MM	30	None	None
	Patient 4	MM	61	None	IVIG, 68

	Patient 5	MM	29	BCMA, 3	IVIG, 112
	Patient 6	MM	29	Daratumumab, 3	IVIG, 117
	Patient 7	MM	30	Daratumumab, 5	IVIG, 47
	Patient 8	MM	22	None	None
	Patient 9	MM	22	None	None
**CD19**					
	Patient 1	Lymphoma	33	Polatuzumab, 1	None
	Patient 2	Lymphoma	28	Autologous HCT, 7	None
	Patient 3	Lymphoma	32	None	None
	Patient 4	Lymphoma	36	None	None
	Patient 5	Lymphoma	27	Ibrutinib, 1	IVIG, 121
	Patient 6	Lymphoma	30	None	None
	Patient 7	Lymphoma	29	None	IVIG, 59
	Patient 8	Lymphoma	26	None	None
	Patient 9	Lymphoma	30	None	IVIG, 28
	Patient 10	Lymphoma	35	Autologous HCT, 8	None
	Patient 11	Lymphoma	30	None	IVIG, 28
	Patient 12	Lymphoma	21	None	None
	Patient 13	Lymphoma	27	None	None
	Patient 14	Lymphoma	27	None	IVIG, 155

Table Legend: MM=multiple myeloma; Sample timing refers to number of weeks between CAR-T infusion and the post-infusion sample being drawn; Prior therapy lists additional

immunomodulatory therapies given in the year prior to initial sample collection, and the number of months between administration of the therapy and collection of the initial sample, excluding the pre-CAR-T conditioning chemotherapy which consists of cyclophosphamide and fludarabine for every patient; Concurrent treatment lists the number of days prior to the second sample being collected when treatment was administered, only IVIG is listed because no other B cell or antibody modulating therapies were given.

Supplemental Table 6: Multiplex Bead-based Protein Array Antigen Panel

Antigen	Antigen Vendor	Antigen Catalog #
IL-1A	Peprotech	200-01A
IL-2	Peprotech	200-02
IL-4	Peprotech	200-04
IL-6	Peprotech	200-06
IL-7	Prospec	CYT-214
IL-11	Peprotech	200-11
IL-15	Peprotech	200-15
IL-17A	Peprotech	200-17
IL-17F	Peprotech	200-25
IL-21	Peprotech	200-21
IL-22	Peprotech	200-22
IL-23	Peprotech	200-23
IL-31	Peprotech	200-31

Influenza A HA (H1)	Kim Lab - Stanford	N/A
Haemophilus Influenzae B	Creative Diagnostics	DAGHIB002
Influenza A HA (H3)	Kim Lab - Stanford	N/A
RSV-F Protein	Sino	11049-V08B
EBV p18	Prospec	EBV-273
EBV EA-D	MyBioSource	MBS319448
HAV VP1	Creative Diagnostics	DAG2415
EBV EBNA-1	Abcam	ab138345
EBV EA	Prospec	EBV-272
HBsAg	MyBioSource	MBS142509
Varicella Zoster Glycoprotein E	MyBioSource	MBS553197
EV71 VP1 Capsid Protein	BioMART	Custom Request
Mumps Nucleoprotein	Prospec	MMP-001
Rubella E1	Prospec	RUB-291
Measles Nucleoprotein	MyBioSource	MBS319759
AcmNPV gp64	Sino Biological	40496-V08B
Rhinovirus VP1	MyBioSource	MBS1220686

Parainfluenza Hemagglutinin-neuraminidase	Sino Biological	40629-V07B
HSV glycoprotein G	MyBioSource	MBS145445
CMV gB	Prospec	CMV-211
HMPV Glycoprotein G	Sino Biological	40791-V08H
Anti-Human IgG Fc fragment Spesific	Jackson	109-005-008
Anti-Human IgG F(ab') fragment specific	Jackson	109-005-006
Anti-Human IgG (H+L)	Jackson	109-005-003
Human IgG from serum	Sigma	I4506
PDC-E2	Diarect	A17901
TPO	Diarect	A12101
TG	Diarect	A12201
CXCL10	Peprotech	300-12
CXCL9	Peprotech	300-26
MIP-1alpha (CCL3)	Peprotech	300-08
CXCL13	Peprotech	300-47
CXCL16	Peprotech	300-55
CXCL5	Peprotech	300-22
CCL21	Peprotech	300-35A
CXCL8	Peprotech	200-08

CCL22	Peprotech	300-36
CCL19	Peprotech	300-29B
CCL25	Peprotech	300-45
CXCL17	Prospec	CHM-024
IFN-alpha1	Prospec	CYT-291
IFN-alpha2	R&D	11101-2
IFN-alpha6	Origene	TP760329
IFN-alpha7	Prospec	CYT-196
IFN-alpha8	Sino	10347-H08H
IFN-alpha10	Sino	10349-H08H
IFN-beta	Peprotech	300-02BC
IFN-epsilon	R&D	9667-ME-025/CF
IFN-omega	Peprotech	300-02J
IFN-gamma	Peprotech	300-02
IFN-lambda1	Peprotech	300-02L
IFN-lambda2	Peprotech	300-02K
C3	Complement Tech	A113
C1q	EMD	204876
FGF7	Peprotech	100-19
GM-CSF	Peprotech	300-03
LIF	Peprotech	300-05
PDGFBB	Peprotech	100-14B
VEGFB	Peprotech	100-20B

TNF-alpha	Peprotech	300-01A
IL1RA	Peprotech	200-01RA
ACE2	Sino biological	10108-H05H
Proteinase 3	Diarect	A18601
BPI	Sigma	SRP6307
IFN-lambda3	R&D	5259-IL-025/CF
IL-33	Peprotech	200-33
CCL26	Peprotech	300-48

Supplemental Table 7: Additional BCMA Patient Demographics

Patient	Cohort	Age Range (years)	Sex	Race/Ethnicity
Patient 10	BCMA	51-55	Female	White
Patient 11	BCMA	61-65	Male	White
Patient 12	BCMA	51-55	Male	Not reported
Patient 13	BCMA	56-60	Male	White

Supplemental Table 8: Additional BCMA Sample Details

CAR-T	Patient	Malignancy	Post-CAR-T Sample Timing (weeks)	Prior therapy (months pre-CAR-T)	Concurrent treatment (days prior to second sample)
**BCMA**					
	Patient 10	MM	27	None	None

	Patient 11	MM	24	None	None
	Patient 12	MM	27	None	None
	Patient 13	MM	22	Elotuzumab, 4	None

Table Legend: MM=multiple myeloma; Sample timing refers to number of weeks between CAR-T infusion and the post-infusion sample being drawn; Prior therapy lists additional immunomodulatory therapies given in the year prior to initial sample collection, and the number of months between administration of the therapy and collection of the initial sample, excluding the pre-CAR-T conditioning chemotherapy which consists of cyclophosphamide and fludarabine for every patient.
